# Intricate diagnosis due to falsely elevated testosterone levels by immunoassay

**DOI:** 10.1007/s12020-025-04191-1

**Published:** 2025-02-13

**Authors:** Benedetta Gardini, Marta Bondanelli, Alessio Cariani, Maria Chiara Zatelli, Maria Rosaria Ambrosio

**Affiliations:** 1 Section of Endocrinology, Geriatrics and Internal Medicine, Department of Medical Sciences, University of Ferrara, Ferrara, Italy; 2 Chemical-Clinical Analysis Laboratory, “S. Anna” University Hospital of Ferrara, Ferrara, Italy

**Keywords:** Testosterone, Laboratory interferences, Heterophilic antibodies, Hormone immunoassay

## Abstract

**Purpose:**

Hormone immunoassay may be subject to interferences and, although rarely reported, this can occur for testosterone too. This work is aimed to highlight the importance of considering possible analytical interferences when the biochemical data doesn’t match with the clinical picture.

**Methods:**

We report three cases of insidious diagnosis due to laboratory interference in testosterone immunoassay, and we provide a brief literature review on this issue.

**Results:**

We found falsely high testosterone levels due to the presence of heterophilic antibodies, leading to unnecessary investigations, misdiagnosis and inappropriate treatments.

**Conclusion:**

The detection of elevated testosterone levels on immunoassay not corresponding to clinical findings need to be confirmed by liquid chromatography-tandem mass spectroscopy, prior to escalation of complex diagnostic investigation and care.

## Introduction

The most accurate and specific method for assessing total testosterone (TT) concentration is liquid chromatography-tandem mass spectroscopy (LC-MS/MS). However, due to its complexity and cost, LC-MS/MS is not widely adopted, and immunoassays (competitive or sandwich) are commonly used [1–3].

Although less common than other hormones, T immunoassay may suffer from interfering factors, either endogenous or exogenous, including blood proteins and drugs with high structural similarity to T [4]. Moreover, endogenous antibodies may cause misdiagnosis due to their cross-reactivity with the immunoassay antibodies, including heterophile antibodies (HBA), human anti-animal, autoimmune, and other nonspecific antibodies, as well as rheumatoid factors [5]. These pitfalls are more frequent in females because of the very low circulating T concentrations [1].

Therefore, clinicians should be suspicious when a discrepancy between clinical picture and laboratory test exists.

We present cases of insidious diagnosis due to laboratory interference in T immunoassay and we revised the literature on this issue.

## Methods and results

A 57-year-old woman arrived at our clinic for a picture of hyperandrogenism worsened after menopause (occurred at 51 years). She showed hirsutism (Ferriman Gallwey-index: 12) and alopecia (Ludwig scale: II-1), associated with high TT plasma level (>3 ng/ml; v.n: 0,10–0,75), confirmed on three different measurements by the same method (Chemiluminescent ImmunoAssay, CLIA, Access Beckman Coulter’ kit on the DXI 800 Beckman Coulter instrument).

Biochemical evaluation and imaging ruled out the major causes of hyperandrogenism, such as Cushing syndrome, 21-hydroxylase deficiency, adrenal mass and ovarian virilizing tumors. Moreover, the patient had hypertension well controlled by therapy and obesity (BMI = 37 Kg/m^2^) without weight changes in the last period. She also showed impaired glucose tolerance to oral glucose tolerance test with normal HOMA-index (<2,5).

Therefore, ovarian hyperthecosis was suspected and the patient received GnRH agonist treatment (Triptorelin 3,75 mg i.m every 28 days) for 3 months without clinical or biochemical response: gonadotropin levels were suppressed, but TT levels remained unchanged. Therefore, the suspicion of ovarian hyperthecosis was not confirmed. Due to a small likely benign ovarian lesion at pelvic MRI, we discussed with gynecologists the possibility of an ovarian tumor and its surgical treatment. Before ovariectomy, TT was measured by a different immunoassay (CMIA, Chemiluminescent Microparticle Immunoassay, ARCHITECT, Abbott), resulting in the normal range (TT = 0.33 ng/ml, v.n: 0,09–0,37 in females > 50 years). TT assay was repeated using CLIA kit previous treatment with the HBA reagent ‘HBT (Heterophilic Blocking Tube) Scantibodies’ resulting in final TT level of 0.12 ng/ml. Selective LC-MS/MS confirmed normal TT level (0.16 ng/ml).

We also report the case of a 67-year-old man followed at our clinic for non-secreting pituitary macroadenoma, surgically treated five years before. The patient showed high gonadotropin levels (LH = 13.8 mU/ml, v.n 1.2–8.6; FSH = 52.5 mU/ml, v.n 1.3–19.3) with TT in the upper range of normal (6.61 ng/ml, v.n 1.75–7.81). He didn’t report sexsual dysfunction, headache and/or visual field abnormalities, and the other hormonal tests were normal. Gadolinium pituitary MRI ruled out the possibility of a gonadotropinoma. Moreover, testicular ultrasonography was negative and Gallium-68 somatostatin receptor positron emission tomography did not indicate ectopic source of gonadotropins; in addition, PSA was suppressed. The patient was previously treated with an antiandrogen drug (bicalutamide) for prostate cancer, but the probability of interference was ruled out because this therapy was stopped four years before.

The dissociation between clinical and laboratory findings suggested the possibility of interference in the TT assay. Therefore, the blood sample was treated with HBT Scantibodies using the Beckman CLIA kit and TT levels resulted 6.29 ng/ml and 14.20 ng/ml, before and after treatment, respectively. The last value was confirmed by a second measurement and on a 1/5 dilution, suggesting HBA interference. To clarify these data, TT was measured using the LC-MS/MS method resulting in the low normal range (TT = 3.00 ng/ml), consistent with the clinical picture of subclinical hypergonadotropic hypogonadism.

**Fig. 1:**
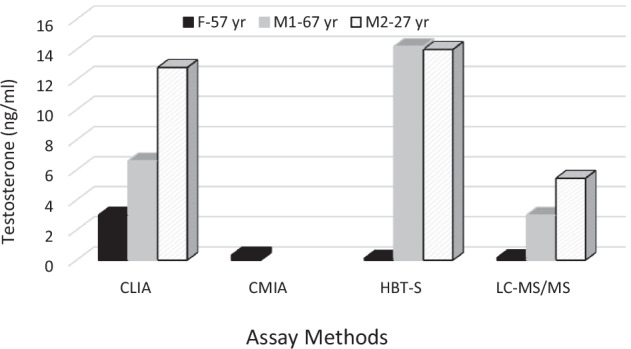
Testosterone levels on different assays. CLIA chemiluminescent immunoassay, CMIA chemiluminescent microparticle immunoassay, HBT-S heterophilic blocking tube scantibodies, LC-MS/MS liquid chromatography-tandem mass spectroscopy

We also report the case of a young man (27 years old) arrived at our clinics for a picture of hypertension and decreased libido. We excluded causes of secondary hypertension and we found high TT levels in two samples (TT 12.8 ng/ml, and 10.33 ng/ml), associated with high free calculated T level (348 pg/ml; v.n 15–200), and with normal SHBG and gonadotropin (LH 3.2 mU/ml, FSH 1.5 mU/ml) levels. Testicular echography was normal. In the suspicion of analytical interference, we measured TT after treatment with HBT Scantibodies resulting 14 ng/ml (Beckman CLIA kit). The discrepancy of the data led us to assess TT with LC-MS/MS method, with final result of normal TT level (5.45 ng/ml) (Fig. 1).

## Discussion

Our data indicate that HBA, although rarely, can interfere with T immunoassay measurement, leading to false-positive and/or false-negative results that could trigger unnecessary investigations, misdiagnosis and/or inappropriate treatments.

HBA are produced against red blood cell proteins of diverse animal species that arise naturally or in response to various external stimuli, such as viral or bacterial infections. They can bind non-specifically and non-competitively to other proteins, including antibodies used in laboratory tests. This may cause interference in diagnostic test results, compromising their accuracy. The exposure to certain animals and animal-derived products is a risk factor for producing HBA, but they can also occur during vaccination, antibody-targeted therapies, blood transfusion and autoimmune disease [6, 7]. In our patients only the oldest man had a known risk factor, because he lives with a dog.

The prevalence of HBA ranges from 0.2–3.7% in the general population, with higher rates in sick and hospitalized patients (ranging from 0.2–15%). However, some reports suggest a prevalence of up to 40% in the general population, which may present different exposition to a foreign antigen (for example, vaccination, blood transfusion, antibody-targeted therapies, exposure to animals, etc).

The interferences on immunoassay caused by HBA are more frequent with sandwich assays, but they may be observed with competitive ones, resulting in falsely elevated analyte levels [5]. Therefore, by removing the HBA from the sample, the true value could be obtained. HBA may also cause false depression of serum ACTH, cortisol and thyroglobulin causing diagnostic difficulties [8].

This phenomenon is quite frequent in certain hormone assays (such as TSH and thyroid hormones) and quite rare in others, such as T. Indeed, rare cases of falsely elevated TT levels due to HBA are reported in women and adolescent. Moreover, elevated TT levels on immunoassay due to heterophile interference have been described in a patient with metastatic prostate cancer, initially thought to be refractory to medical castration, who underwent bilateral orchiectomy [1, 7, 9–11].

In addition, falsely elevated TT levels have also been identified in on case of anti-testosterone antibody presence, in rare cases of gammopathies (due to IgG interference), and in patients taking biotin (due to the use of biotinylated reagents). TT levels may also be overestimated due to cross-reactivity with other steroid hormones or medications with similar structural activity (Table 1) [4, 5, 9–19].

**Table 1:** Interfering factors with testosterone immunoassay testing

References	Compounds/Interfering Factor	Immunoassay	Interference Type	T Level
Kuwahara A et al. [12]	Antitestosterone autoantibody	RIA	Analytical interference	Falsely elevated
Cresta F et al. [10]; Sarkar A et al. [11]	Heterophilic antibodies	ECL (Roche, Modular analytics E170 analyzer); ECL	Analytical interference	Falsely elevated
Moerman A et al. [13]	Biotin	Ortho; Roche; Siemens	Analytical interference	Falsely elevated
Kane J et al. [4] Sofronescu AG et al. [14]	Danazole Mifepristone Asfotase alfa	ECL (Bayer Advia Centaur and Roche Elecsys/E170 Modular; Beckman Access and DPC Immulite 2000) RIA, ECL (Beckman Coulter DxI 800)	Analytical interference	Overestimated Falsely reduced
Langlois F et al. [15]	Monoclonal IgG hypergammaglobulinemia	ECL (Roche)	Multiple analytic interference	Falsely elevated
Ramaeker D et al. [16]	Polyclonal gammopathy in acute myelogenous leukemia	ECL (Immulite 2000)	Analytical interference	Falsely elevated
Jansen HI et al. [17]	Chronic renal failure in hemodialysis	ECL (Roche; Immulite 2000)	Not defined interfering factor(s)	Overestimated
Ghazal K et al. [9]	DHEA-S	ECL (Abbott Architect)	Crossreactivity	Overestimated
Krasowski MD et al. [18]; Dasgupta A. [5]	Anabolic steroids (boldenone, 19-norclostebol, dianabol, methyltestosterone, norethindrone, normethandrolone, and 11β-hydroxytes- tosterone)	ECL (Roche)	Crossreactivity ≥5%	Overestimated
Heijboer AC et al. [19]	Oral Contraceptive	RIA	Increased SHBG	Underestimated

*RIA* radioimmunoassay, *ECL* electrochemiluminescence

In our work, HBA interference was detected only with CLIA method. In fact, TT levels were higher than expected, both in women and in men, as measured by CLIA, whereas it was in the normal range when measured by competitive CMIA method in the woman. As previously discussed, serum may contain interference factors that may cause false results depending on different immunoassay. In the present work, CMIA kit did not give a false result probably due to the presence of different amount of blocking reagents against HBA incorporated in the kits. Indeed, given the frequent presence of HBA in the population, in recent years, the main assay kits have been equipped with reagents capable of reducing this interference. HBA are highly heterogeneous and variable in their concentrations among individuals, therefore no blocking reagent can completely ensure protection against such interference [3, 5].

As expected, in our woman a reduction in TT levels was obtained after treating the sample with the HBA reagent ‘HBT Scantibodies’ confirming that the high level was due to assay interference. The data was subsequently confirmed by LC-MS/MS method.

By contrast, both men showed an increase in TT level after treatment with HBA reagent. Rare cases of increased analyte level, rather than decreased, are described in literature after treatment with ‘HBT Scantibodies’ [20]. A possible explanation for this phenomenon is that the use of HBA-blocking agent removes weakly reacting HBA (of the IgG class), while leaving highly reactive specific IgM class antibodies. These specific IgM antibodies may then bind more strongly to the analyte antibody, leading to increased interference in the assay [5]. These data indicate that when the measured value after treatment with HBA reagent differs from the initial value (whether it increases or decreases), further investigations are needed to verify the real values. Only LC-MS/MS method can definitively provide the correct interpretation of T level, by removing these analytical interferences [2, 7]. In fact, also in our man TT levels obtained by LC-MS/MS were consistent with clinical data.

It is known that TT immunoassay measurements can have limited accuracy, especially at low concentrations, and can be subject to interference, while LC-MS/MS method is sensitive and specific, but it is more expensive and less available, therefore excluding its routinary use in all clinical laboratories [2].

When the clinical picture is not supported by the laboratory data, physicians need to consider the potential presence of analytical interference, because misdiagnosis can lead to unnecessary procedures for patients and can limit the quality of life.

In the woman the diagnosis of laboratory interference was insidious because she had clinical manifestations of hyperandrogenism, which led to many diagnostic investigations to exclude any possible organic cause. In fact, the misdiagnosis of a possible ovarian causes of hyperandrogenism led us to suggest an ovariectomy, according with gynecologist. The final finding of normal TT levels indicated that the woman suffered from a common pattern of androgenetic alopecia, which is also frequent in women. Similarly, in literature is reported a case of bilateral orchiectomy performed in a man with prostate cancer due to false high T level [11].

In our work also the men undergone many diagnostic procedures to exclude organic causes of hypertestosteronemia, causing considerable stress and negative economic impact. LC-MS/MS demonstrated the true TT levels, which were normal in the youngest one and consistent with the clinical picture of subclinical hypergonadotropic hypogonadism still compensated in the oldest one.

## Conclusion

These findings are consistent with interference by HAB in T competitive immunoassay, causing falsely elevated levels and unnecessary diagnostic procedures with negative impact on healthcare and quality of life of the patients. The detection of elevated TT levels on immunoassay not corresponding to clinical findings need to be confirmed by LC-MS/MS, prior to escalation of complex diagnostic investigation and care.

## Data Availability

No datasets were generated or analysed during the current study.
